# Longitudinal association of intensity-specific physical activity and sedentary behavior with dynapenia among older Taiwanese adults

**DOI:** 10.1186/s11556-025-00382-x

**Published:** 2025-11-06

**Authors:** Chih-Ching Chang, Jiaren Chen, Ting-Fu Lai, Jong-Hwan Park, Yung Liao

**Affiliations:** 1 Department of Health Promotion and Health Education, College of Education, National Taiwan Normal University, Taipei, Taiwan; 2 Graduate Institute of Sport, Leisure and Hospitality Management, College of Sports and Recreation, National Taiwan Normal University, Taipei, Taiwan; 3 Health Convergence Medicine Laboratory, Biomedical Research Institute, Pusan National University Hospital, Busan, Republic of Korea; 4 Department of Convergence Medicine, Pusan National University School of Medicine, Yangsan, Republic of Korea; 5 Department of Clinical Bio-Convergence, Graduate School of Convergence in Biomedical Science, Pusan National University School of Medicine, Yangsan, Republic of Korea; 6 Convergence Medical Institute of Technology, Pusan National University Hospital, Busan, Republic of Korea; 7 Faculty of Sport Sciences, Waseda University, Tokorozawa, Japan

**Keywords:** Physical activity intensity, Accelerometer, Handgrip, Muscle weakness

## Abstract

**Background:**

Given that limited research has examined the relationships between lifestyle activities of varying intensities, including moderate-to-vigorous physical activity (MVPA), light physical activity (LPA), and sedentary behavior (SB), and dynapenia, which refers to an age-related decline in muscle function, this study aimed to investigate the longitudinal associations between MVPA, LPA, and SB and the risk of dynapenia among older adults in Taiwan.

**Methods:**

This longitudinal study included older adults aged ≥ 65 years with independent mobility, recruited from the National Taiwan University Hospital. Baseline data were collected from September 2020 to December 2021 and follow-up data were collected until December 2022. Participants wore a tri-axial accelerometer (GT3X + ActiGraph) on the hip for seven consecutive days to evaluate baseline time spent of MVPA (≥ 2020 counts/min), LPA (100–2019 counts/min), and SB (< 100 counts/min). To confirm the dynapenia classification at baseline and follow-up, participants underwent standard assessments, including handgrip dynamometry for muscle strength, bioelectrical impedance analysis for muscle mass, and a 6-m walk test for physical performance. Adjusted binary logistic regression analyses were conducted to examine the association between lifestyle activities and dynapenia risks.

**Results:**

Among 154 participants (mean age 80.3 ± 7.2 years; 53.9% women), 53.9% were classified as having dynapenia at baseline, compared to 55.2% at follow-up. Participants spent an average of 16.9 (± 26.6) min in MVPA, 249.5 (± 85.7) min in LPA, and 604.5 (± 76.4) min in SB daily. The longitudinal analysis results indicated that higher MVPA time was significantly associated with lower odds of dynapenia in both the unadjusted (odds ratio [OR] = 0.625, 95% confidence interval [CI]: 0.466–0.837) and fully adjusted models (OR = 0.578, 95% CI: 0.406–0.823). Each additional 10 min/day of MVPA was associated with 42.2% lower odds of dynapenia in the adjusted model. No significant prospective associations were observed between the LPA or SB time and dynapenia.

**Conclusion:**

This study provides longitudinal evidence that higher MVPA levels are significantly associated with a reduced dynapenia risks among community-dwelling older adults in Taiwan. These findings underscore the importance of promoting MVPA as a part of lifestyle interventions aimed at preserving muscle function and preventing dynapenia in older populations.

## Introduction

Over recent decades, muscle strength has been increasingly recognized as a more reliable indicator of adverse health outcomes than muscle mass alone [1–3]. Recent evidence suggests that reduced muscle strength and/or poor physical performance are key indicators of declining muscle function [4], and are more clinically relevant than low muscle mass alone in predicting functional limitations [5], disability, hospitalization [6], and all-cause mortality [3] among older adults. These findings underscore the importance of maintaining muscle function to support mobility, functional independence, and the quality of life in later years.

Dynapenia, defined as an age-related decline in muscle function in the presence of normal muscle mass [7, 8], is increasingly recognized as a distinct clinical condition in older populations. In contrast to sarcopenia, which involves a decline in both muscle function and mass [9], dynapenia represents an earlier and potentially modifiable stage in the progression of muscle deterioration [4, 10]. Dynapenia has been independently associated with a range of adverse physical and psychological outcomes in older adults, including impaired instrumental activities of daily living [11, 12], increased fall risk [10], depressive symptoms [13, 14], cognitive deterioration [15], and mortality [16, 17], highlighting its significance as a growing public health concern in aging societies. In Taiwan, the proportion of individuals aged 65 years and older is projected to exceed 20% by 2025, indicating the nation’s transition to a super-aged society [18]. Concurrently, community-based studies have reported that 28–31% of older adults have dynapenia [19, 20], suggesting a substantial and possibly escalated burdens on the aging population. Furthermore, longitudinal data have shown that 18% of initially robust older adults developed dynapenia over six-years [21], indicating its progressive course and underscoring the potential for early intervention. This rapid demographic shift and high prevalence of dynapenia in Taiwan highlight the urgent need to identify modifiable behavioral factors and inform the development of timely and targeted interventions.

Physical activity (PA) and sedentary behavior (SB) are key modifiable behavioral determinants associated with muscle function in older adults [22]. Regular PA has been shown to preserve muscle strength and reduce the incidence of mobility impairments and falls [23, 24]. Across the intensity spectrum, both moderate-to-vigorous physical activity (MVPA) and light intensity physical activity (LPA) have been shown to be positively associated with muscle function in older populations [25, 26]. In contrast, prolonged sedentary behavior has been associated with a higher risk of sarcopenia [27, 28]. Taken together, these findings underscore the clinical importance of reducing sedentary time and promoting PA across all intensity levels to attenuate age-related declines in muscle function.

Although the effects of PA and SB on muscle health are well-established, their specific association with dynapenia remains unclear. Previous research linking low PA and excessive SB to higher dynapenia risk often involve populations with chronic conditions or multiple comorbidities [29–31], limiting the applicability of these findings to healthier, community-dwelling, older populations. Moreover, most studies have relied on self-reported assessments of PA and SB [32], which are susceptible to recall and reporting biases. Additionally, the predominance of cross-sectional study designs [33] further limits the ability to draw causal inferences regarding the temporal relationships between PA and SB exposure and the risk of dynapenia. To address these research gaps, this study employed accelerometer-based assessments to objectively quantify the daily time spent in MVPA, LPA, and SB, and adopted a longitudinal design to examine the prospective associations between these behavioral exposures and the onset of dynapenia among community-dwelling older adults in Taiwan. This methodological approach provides a more robust and temporally grounded understanding of modifiable PA-related behavioral factors and supports the development of targeted interventions for the prevention of dynapenia in older adults.

Therefore, this study aimed to investigate the longitudinal associations between accelerometer-measured MVPA, LPA, and SB and the risk of dynapenia in older Taiwanese adults.

## Materials and methods

### Study design

In this longitudinal study, a convenience sample of older adults aged 65 years and older with independent mobility was recruited from the National Taiwan University Hospital through two pathways: individuals undergoing routine health checkups and those referred by physicians from the Department of Geriatrics and Gerontology outpatient clinic. Prior to data collection, all participants were fully informed of the study’s purpose and procedures, and written informed consent was obtained to ensure the protection of the participants rights and autonomy. Each participant received a US$7 convenience store voucher upon completion of the study as a token of appreciation. This study was approved by the Research Ethics Committee of the National Taiwan University Hospital (REC number: 202008046RINC).

Baseline data were collected from September 2020 to December 2021, and participants were followed up after an interval of at least one year, with follow-up data collection completed by December 2022. At baseline, interviewer-administered questionnaires were used to obtain information on their sociodemographic characteristics, health status, and health behaviors. Subsequently, participants underwent standardized assessments for dynapenia classification, including handgrip strength (HGS) for muscle strength, 6-m gait speed for physical performance, and bioelectrical impedance analysis (BIA) for muscle mass. Dynapenia classification was performed according to the cutoff values recommended in the 2019 consensus of the Asian Working Group for Sarcopenia (AWGS) [9]. Finally, to objectively measure the average time spent in MVPA, LPA, and SB, the participants were instructed to wear a triaxial accelerometer for seven consecutive days. Accelerometer data were collected during the baseline phase of the study. At the one-year follow-up, participants completed the same interviewer-administered questionnaires and standardized assessments to determine their dynapenia status.

### Participants

The sample size was estimated using G*Power software (version 3.1.9.7) [34]. A minimum of 131 participants were required to detect a statistically significant effect in a binary logistic regression model, assuming an alpha level of 0.05, and a power of 0.80. To ensure an adequate sample size, 291 individuals who expressed willingness to participate in the study were initially recruited. Subsequently, the following exclusion criteria were applied: [1] missing questionnaire responses, incomplete measurements required for dynapenia classification, or insufficient accelerometer data (*n* = 73); and [2] participants diagnosed with sarcopenia to avoid misclassification between dynapenia and non-dynapenia (*n* = 7). Thus, 211 participants were included in the baseline analysis.

**Fig. 1:**
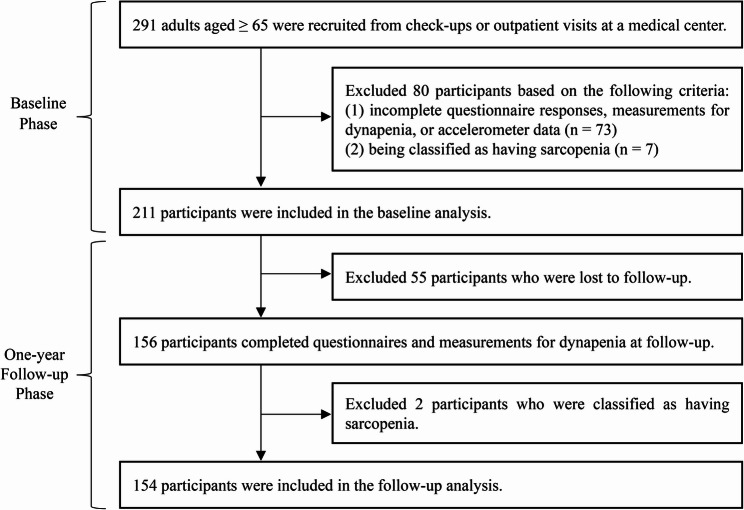
Flowchart of participant recruitment and follow-up

At the one-year follow-up, 55 participants were lost to follow-up. The remaining 156 participants completed both the questionnaires and standardized assessments for dynapenia. Among them, 2 participants were classified as having sarcopenia and excluded to maintain consistency with the baseline analysis criteria. A total of 154 participants were thus included in the final analysis (Fig. 1).

### Measures

#### Intensity-specific PA and SB

The daily time spent on intensity-specific PA and SB was objectively assessed using a waist-worn triaxial accelerometer (GT3X + ActiGraph, Pensacola, FL, United States). The participants were instructed to wear the device on their right hip continuously for seven consecutive days during both waking and sleeping hours, except during bathing or other water-based activities. Accelerometer data were collected at a sampling frequency of 30 Hz, recorded in 60-s epochs, expressed in counts per minute (cpm), and processed using the ActiLife software (version 6.0; ActiGraph LLC). A valid day was defined as having at least 10 h of wear time, and participants with a minimum of four valid days, including at least three weekdays and one weekend day [35, 36], were included in the analysis. PA intensity was classified using validated thresholds: MVPA was defined as ≥ 2,020 cpm, LPA as 100–2,019 cpm, and SB as < 100 cpm [35]. The average daily time spent in each category was calculated across valid days.

#### Dynapenia

Dynapenia was operationally defined as the presence of low muscle strength and/or poor physical performance in the context of normal muscle mass [7]. This study adopted the diagnostic algorithm and cutoff thresholds recommended by the 2019 AWGS consensus [9]. Standardized assessments included HGS for muscle strength, gait speed over a 6-m distance for physical performance, and appendicular skeletal muscle mass (ASM) measured using BIA. Participants with normal HGS, gait speed, and ASM were classified as not having dynapenia. In contrast, sarcopenia was defined as the co-occurrence of low HGS and poor gait speed, accompanied by low ASM [7]. Participants who met the criteria for sarcopenia were excluded from the analysis to ensure a clear distinction between those with and without dynapenia.

#### HGS assessment

HGS was measured to assess upper limb muscle strength using a hydraulic handheld dynamometer (Jamar Plus + Digital Hand Dynamometer, Lafayette Instrument Company, United States). The participants were instructed to stand upright with their arms relaxed at their sides and squeeze the dynamometer with their dominant hand with maximum effort. Two trials were conducted with a 1-min rest interval between attempts. The highest value recorded (in kg) was used for the analysis. Low muscle strength was defined as HGS < 28.0 kg for men and < 18.0 kg for women [9].

#### Gait speed assessment

Gait speed was assessed to evaluate physical performance using the 6-m walk test. Participants were instructed to walk a straight 6-m path at their usual pace on a flat and unobstructed surface, with trained assessors walking alongside to ensure safety and adherence to the protocol. Gait speed was measured using a handheld stopwatch, and the average speed from the two trials, recorded in meters per second (m/s), was used for the analysis. Poor physical performance was defined as a gait speed < 1.0 m/s for both men and women [9].

#### ASM assessment

ASM was assessed using a dual-frequency body composition analyzer (DC-430MA; TANITA Corporation, Tokyo, Japan) [37]. The participants were instructed to stand upright and barefoot on the analyzer platform, with both hands gripping the handles and remaining still until the measurement was completed. ASM was automatically calculated using the device and expressed in kilograms per square meter (kg/m²) as the ASM mass index. Low muscle mass was defined as ASM < 7.0 kg/m² for men and < 5.7 kg/m² for women [9].

#### Covariates

Several covariates were included based on previous literature and their potential associations with dynapenia [14, 38–42]. These covariates were obtained at baseline via interviewer-administered questionnaires, included age (65–74 years or ≥ 75 years), sex (male or female), educational level (less than university vs. university or above), number of self-reported comorbidities (< 4 or ≥ 4 conditions), smoking status, and alcohol consumption (categorized as “yes” or “no” based on use in the past year). The body mass index (BMI; kg/m²) was calculated by dividing weight in kilograms by height in meters squared. Nutritional risk was assessed using the Mini-Nutritional Assessment–Short Form, with scores ≤ 11 indicating nutritional risk and scores > 11 indicating normal nutritional status [43]. The total accelerometer wear time (min/day) was derived from ActiGraph data, averaged across valid wear days, and included as a covariate to control for individual differences that could bias the estimates of PA and SB [44].

### Statistical analysis

IBM SPSS (version 23.0; SPSS Inc., Chicago, IL, USA) was used for all the statistical analyses. Descriptive statistics were used to summarize the baseline sociodemographic characteristics and dynapenia status at both baseline and follow-up. Categorical variables were presented as frequencies and percentages. The baseline MVPA, LPA, and SB times were treated as continuous variables and reported as means and standard deviations. The normality of difference scores between baseline and follow-up was assessed using the Shapiro-Wilk test. Group-level differences in MVPA, LPA, and SB time between participants with and without dynapenia at baseline were examined using the Mann–Whitney U test. The Wilcoxon signed-rank test was applied to examine differences in muscle strength, gait speed, and muscle mass between baseline and one-year follow-up within each sex group. Finally, in the main analysis, binary logistic regression models were used to examine the prospective associations between intensity-specific PA, SB, and dynapenia. For the prospective analysis, dynapenia status at one-year follow-up served as the outcome variable, with baseline dynapenia status included as a covariate to assess the longitudinal relationship. The independent variables were MVPA time (per 10 min/day), LPA time (per 1 min/day), and SB time (per 30 min/day). These units were defined to facilitate interpretation of the regression coefficients, enhance the comparability of effect sizes, and to avoid generating excessively small values that would be difficult to interpret. Specifically, a 10-minute/day unit was used for MVPA to allow comparison with a previous accelerometer-based study [45]. LPA and SB accounted for large proportions of daily activity in our sample (approximately 250 min/day for LPA and over 600 min/day for SB). Therefore, different units were selected to maintain interpretability and to reflect practical behavioral change targets, such as reducing sedentary time by half an hour per day. Two models were constructed for each analysis: Model 1 was unadjusted and Model 2 was adjusted for potential covariates, including age, sex, educational level, BMI, number of comorbidities, smoking status, alcohol consumption, nutritional status, and total accelerometer wear time. Adjusted odds ratios (ORs) with 95% confidence intervals (CIs) were estimated, and statistical significance was set at *p* < 0.05.

## Results

A total of 154 individuals were included in both the baseline and one-year follow-up analyses. Table 1 summarizes the baseline characteristics of the participants. Overall, 53.9% of the participants were women, with a mean age of 80.3 (± 7.2) years, and 50.6% of participants had an educational level below university. Regarding health status, 48.1% of the participants had a normal BMI, 84.4% had normal nutritional status, and 89.0% reported fewer than four comorbid conditions. With respect to lifestyle behaviors, most participants had not used tobacco (92.2%) or alcohol (89.6%) during the past week. On average, participants engaged in 16.9 (± 26.6) min/day of MVPA, 249.5 (± 85.7) min/day of LPA, and 604.5 (± 76.4) min/day of SB. Notably, 83 participants (53.9%) were classified as having dynapenia at baseline, compared to 85 participants (55.2%) at follow-up.

**Table 1:** Baseline characteristics of participants (*n* = 154)

Categorical variables	Total	
	*N*	%
**Age**		
65–74	42	27.3
≥ 75	112	72.7
**Sex**		
Female	83	53.9
Male	71	46.1
**Educational level**		
Less than university	78	50.6
University or above	76	49.4
**Smoking**		
No	142	92.2
Yes	12	7.8
**Alcohol drinking**		
No	138	89.6
Yes	16	10.4
**Comorbidity (No. of conditions)**		
< 4	137	89.0
≥ 4	17	11.0
**BMI**		
Underweight	2	1.3
Normal	74	48.1
Overweight	47	30.5
Obesity	31	20.1
**Nutritional status**		
Normal	130	84.4
At risk	24	15.6
**Dynapenia in baseline**		
No	71	46.1
Yes	83	53.9
**Dynapenia in follow-up**		
No	69	44.8
Yes	85	55.2
**Continuous variables**	**Mean**	**SD**
MVPA time (min/day)	16.9	26.6
LPA time (min/day)	249.5	85.7
SB time (min/day)	604.5	76.4
Total accelerometer wear time (min/day)	870.9	80.8

*Abbreviations*: *SD *Standard deviation, *BMI B*ody mass index, *MVPA *Moderate-to-vigorous physical activity, *LPA *Light physical activity, *SB *Sedentary behavior

Table 2 presents group-level comparisons of time spent in intensity-specific PA and SB between participants with and without dynapenia at baseline. Participants with dynapenia spent significantly less time in MVPA (8.59 min/day vs. 26.71 min/day, *p* < 0.001) and LPA (227.80 min/day vs. 274.79 min/day, *p* < 0.001), and more time in SB (621.50 min/day vs. 584.66 min/day, *p* = 0.009) than those without dynapenia.

**Table 2:** Comparison of time spent in intensity-specific physical activity and sedentary behavior between participants with and without dynapenia at baseline

Variables	Non-dynapenia (*n* = 71)		Dynapenia (*n* = 83)		*p*-value
	Mean	SD	Mean	SD	
MVPA time (min/day)	26.71	34.12	8.59	13.30	< 0.001***
LPA time (min/day)	274.79	69.53	227.80	92.37	< 0.001***
SB time (min/day)	584.66	75.09	621.50	73.76	0.009**

*Abbreviations*: *SD *Standard deviation, *MVPA *Moderate-to-vigorous physical activity, *LPA *Light physical activity, *SB *Sedentary behavior

^**^p < 0.01; ^***^p < 0.001

Table 3 presents sex-stratified results for muscle strength, gait speed, and muscle mass at baseline and one-year follow-up. Among female participants, no significant differences were observed between baseline and follow-up values for muscle strength (17.70 kg vs.17.73 kg), gait speed (1.02 m/s vs. 1.25 m/s), or muscle mass (6.78 kg/m² at both time points). In contrast, among male participants, muscle strength significantly increased from 28.59 kg at baseline to 29.42 kg at follow-up (*p* = 0.03), and gait speed also improved from 1.19 m/s to 1.55 m/s (*p* = 0.04). No significant differences in muscle mass was observed between the two time points.

**Table 3:** Muscle strength, gait speed, and muscle mass by sex at baseline and follow-up

Variables	Female					Male				
	Baseline		Follow-up		*p*-value	Baseline		Follow-up		*p*-value
	Mean	SD	Mean	SD		Mean	SD	Mean	SD	
Muscle strength (kg)	17.70	4.75	17.73	5.20	0.72	28.59	7.29	29.42	8.07	0.03*
Gait speed (m/s)	1.02	0.36	1.25	1.14	0.33	1.19	0.36	1.55	1.27	0.04*
Muscle mass (kg/m²)	6.78	0.72	6.78	0.78	0.91	9.09	1.11	9.02	1.20	0.28

*Abbreviations*: *SD *Standard deviation. ^*^p < 0.05

Table 4 presents the prospective analysis examining the association between baseline intensity-specific PA, SB, and dynapenia at one-year follow-up. Model 1 was unadjusted, whereas Model 2 was adjusted for covariates and baseline dynapenia status. The longitudinal analysis demonstrated that higher engagement in MVPA was significantly associated with lower odds of dynapenia. In the unadjusted model (Model 1), each additional 10 min/day of MVPA was associated with 37.5% lower odds of dynapenia (OR = 0.625, 95% CI = 0.466–0.837, *p* = 0.002). This association remained significant in fully adjusted Model 2, where each additional 10 min/day of MVPA was associated with 42.2% lower odds of dynapenia (OR = 0.578, 95% CI: 0.406–0.823, *p* = 0.002). In contrast, no significant prospective associations were observed between the LPA or SB time and dynapenia in either model.

**Table 4:** Prospective associations of intensity-specific physical activity and sedentary behavior with dynapenia at follow-up (*n* = 154)

Variables	Model 1			Model 2		
	OR	95%CI	*p*-value	OR	95%CI	*p*-value
MVPA time (per 10 min/day)	0.625	(0.466, 0.837)	0.002**	0.578	(0.406, 0.823)	0.002**
LPA time (per 1 min/day)	0.999	(0.994, 1.004)	0.650	1.000	(0.993, 1.007)	0.999
SB time (per 30 min/day)	1.012	(0.863, 1.186)	0.883	0.968	(0.774, 1.211)	0.777

*Abbreviations*: *OR *Odds ratio, *CI *Confidence interval, *MVPA *Moderate-to-vigorous physical activity, *LPA *Light physical activity, *SB *Sedentary behavior

Model 1 was unadjusted; Model 2 was adjusted for age, sex, educational level, BMI, comorbidity, smoking status, drinking, nutritional status, total accelerometer wear time, and status of dynapenia in the baseline

^**^p < 0.01

## Discussion

To our knowledge, this is the first longitudinal investigation study to examine the associations between objectively measured intensity-specific physical activity and sedentary behavior with the risk of dynapenia among community-dwelling older adults in Taiwan. The findings revealed that greater time spent on MVPA was significantly associated with a lower risk of dynapenia, even after adjusting for multiple potential covariates. In contrast, no significant longitudinal associations were observed for LPA or SB, underscoring the critical role of MVPA in preserving muscle function in older adults.

Our findings are consistent with previous research indicating that older adults with dynapenia spend less time in MVPA and LPA and more time in SB compared to those without dynapenia [33]. Furthermore, several studies have also reported inverse associations between PA, particularly MVPA, and the risk of dynapenia in older adults [29, 30, 32, 33]. In Asia, prior studies have shown an inverse association between PA and dynapenia among older adults with chronic conditions such as chronic obstructive pulmonary disease [30], cardiovascular disease, diabetes mellitus, or chronic liver disease [29]. In contrast, our study focused on relatively healthy, community-dwelling older adults, thereby extending the existing evidence to a broader and more representative population in the region. Focusing on this population enhances the external validity of our findings, emphasizes their potential to benefit from lifestyle-based interventions, and provides actionable evidence to inform community-level strategies.

The protective effect of MVPA against dynapenia can be attributed to multiple interrelated mechanisms. Physiologically, MVPA improves cardiorespiratory fitness and oxygen delivery to the peripheral tissues, thereby enhancing mitochondrial function and skeletal muscle energy metabolism in older adults [26]. These adaptations help prevent declines in muscle function observed with advancing age [46], which is a key determinant of dynapenia. In addition to metabolic adaptations, regular physical training, such as moderate-to-vigorous aerobic and functional activities, may help preserve neuromuscular health in older adults, particularly by promoting muscle fiber reinnervation and maintaining neuromuscular junction integrity [47, 48]. These neuromuscular adaptations are critical as they contribute to the preservation of muscle strength and physical performance, which in turn supports functional mobility and are critical for preventing dynapenia [4, 49]. Moreover, MVPA may exert protective effects by modulating chronic low-grade inflammation, as reflected by the elevated levels of inflammatory markers such as C-reactive protein and fibrinogen [50]. These anti-inflammatory responses help preserve muscle protein integrity, prevent catabolic signaling, and maintain regenerative capacity of muscle tissue [51, 52], thereby attenuating muscle function decline and reducing the risk of dynapenia in older adults. Beyond the physiological mechanisms, MVPA may also help prevent dynapenia through psychological and behavioral pathways, and has been associated with reduced psychological distress and an improved quality of life in older adults [53]. These psychological benefits, in turn, may enhance motivation and self-efficacy to remain active [54], thereby promoting sustained engagement in PA and preserving muscle function.

However, some findings from previous studies contradict the results of the present study, as no significant associations were found between LPA or SB and the risk of dynapenia. Several factors may explain this discrepancy. First, many previous studies have relied on self-reported physical activity measures, such as the International Physical Activity Questionnaire [32, 33] and Global Physical Activity Questionnaire [29, 30], which are susceptible to recall and social desirability biases and may fail to accurately capture activity intensity or SB [55, 56]. In contrast, our study utilized accelerometer-based assessments, providing more precise and objective estimates of activity intensity and duration [57, 58], potentially contributing to the observation of non-significant associations. Second, differences in sample characteristics across the studies may have influenced the observed associations. Existing evidence suggests that the relationship between PA and dynapenia may vary based on the older adults’ comorbidity status [29], suggesting that our relatively healthy, community-dwelling population may have been less responsive to the effects of LPA. Although SB was not significantly associated with dynapenia in our study, the robust inverse association observed with MVPA underscores its critical role. This finding aligns with those of previous longitudinal studies suggesting that the health risks associated with prolonged SB may be mitigated or even offset by sufficient levels of MVPA [59–61]. Therefore, our results further emphasize that targeted interventions should focus on increasing MVPA rather than merely reducing SB [28] to maintain muscle function and prevent dynapenia in older adults. Furthermore, future studies should incorporate longer follow-up durations and more refined assessments of SB patterns to better evaluate long-term effects on muscle health.

The prevalence of dynapenia at baseline exceeded half of the study sample, reaching 53.9%, which may be attributed to several underlying contributors. First, the participants were older adults with a mean age of 80.3 years. This advanced age likely contributed to the high prevalence of dynapenia, as muscle function declines progressively with age [16, 62]. In particular, the rate of decline in muscle strength accelerates more rapidly than the loss of muscle mass after the age of 75 [63]. Second, recruitment from health examination centers and outpatient clinics may have led to an overrepresentation of individuals with existing health conditions or reduced physical function. Third, previous studies suggest that older Taiwanese adults engage in relatively low levels of MVPA [45] or vigorous-intensity physical activity [64], further highlighting their susceptibility to dynapenia. These contextual factors should be considered when assessing the representativeness and generalizability of the findings.

Notably, this study applied the cutoff thresholds recommended by the 2019 AWGS to classify dynapenia and sarcopenia. After one year of follow-up, two participants with dynapenia at baseline progressed to sarcopenia. Although these cases were excluded from the main analysis to maintain consistency when comparing the dynapenia and healthy groups, their progression illustrates the potential of dynapenia as an early indicator of sarcopenia. This underscores the importance of early detection and timely intervention. The adoption of the AWGS framework enhanced the clinical relevance and regional applicability of our findings by applying validated criteria tailored to older Asian populations. This observation further supports the need for longitudinal research to better examine the trajectory of muscle decline and develop strategies for its prevention and early management.

Although this study had several methodological strengths, it also had some limitations. First, the one-year follow-up duration may not be sufficient to capture the full trajectory of long-term muscle decline, particularly considering the potential accelerations in age-related physiological changes among older adults. Notably, the observed improvements in muscle strength and gait speed among older male participants might reflect a survival or participation bias, whereby healthier individuals were more likely to return for follow-up, rather than indicating a genuine reversal of decline in the general population. Second, potential selection bias may be linked to the recruitment strategy. Sourcing participants from outpatient clinics and health examination centers likely attracted a cohort that was healthier or more health-conscious than the general older adult population in Taiwan. Consequently, the generalizability of our findings to a broader population may be limited. Third, based on the study design and considerations of participant burden, this study aimed to examine the longitudinal association of intensity-specific PA and SB with dynapenia by controlling for baseline exposure, a commonly adopted approach in prospective cohort studies. Consequently, the assessment of lifestyle behaviors and accelerometer-measured physical activity solely at baseline precluded the analysis of how changes in these factors during the follow-up period may have influenced the outcomes. Fourth, the waist-worn triaxial accelerometer is a valuable tool for objectively capturing physical activity patterns in older adults [65], particularly when compared to self-reported measures [66]. However, its ability to detect upper-limb movements, static postural activities, or water-based activities such as swimming is limited, which may lead to underestimation of LPA or MVPA. Finally, we excluded two participants who developed sarcopenia at follow-up to maintain consistency with the baseline criteria. However, this may have unintentionally excluded cases that reflect the natural progression from dynapenia to sarcopenia. Future studies should consider longer follow-up durations, repeated measurements of physical activity and related behaviors, inclusion of cases progressing from dynapenia to sarcopenia, or broader recruitment strategies to improve generalizability, better capture behavioral and physiological variability over time, and clarify the role of dynapenia as an early transitional stage in muscle decline.

## Conclusions

This study provided longitudinal evidence that higher MVPA levels are significantly associated with lower risks of dynapenia among community-dwelling older adults in Taiwan. These findings underscore the importance of promoting MVPA as a part of lifestyle interventions aimed at preserving muscle function and preventing dynapenia in older populations.

## Data Availability

No datasets were generated or analysed during the current study.
